# Construction and validation of a predictive model for hepatocellular carcinoma based on serum markers

**DOI:** 10.1186/s12876-022-02489-2

**Published:** 2022-09-13

**Authors:** Liming Zheng, Zeyu Huang, Xiaoping Li, Meifang He, Xiaoqin Liu, Guojun Zheng, Xike Zhou, Longgen Liu

**Affiliations:** 1 Clinical Lab, The Third People’s Hospital of Changzhou, Changzhou, China; 2 Institute of Hepatology, The Third People’s Hospital of Changzhou, 300 Lanling Road, Changzhou City, 213001 Jiangsu China; 3 Clinical Lab, Wuxi No 5 People’s Hospital, Wuxi, China

**Keywords:** Hepatocellular carcinoma, Early diagnosis, Non-invasive predictive model

## Abstract

**Background:**

Early hepatocellular carcinoma (HCC) detection with non-invasive biomarkers remains an unmet clinical need. We aimed to construct a predictive model based on the pre-diagnostic levels of serum markers to predict the early-stage onset of HCC.

**Methods:**

A total of 339 HCC patients (including 157 patients from Changzhou cohort and 182 patients from Wuxi cohort) were enrolled in our retrospective study. Levels of 25 baseline serum markers were collected. Propensity score matching (PSM) analysis was conducted to balance the distributions of patients’ gender, age, and the surveillance time between HCC group and control group. Then, Receiver operating characteristic (ROC) and Logistic regression analysis were performed to screen the independent predictive variables and construct a non-invasive predictive model. Subsequently, ROC curve and Kaplan–Meier (K–M) curve were used to evaluate the predictive values of the model. Clinical net benefit of the model was demonstrated by decision curve analysis (DCA) and clinical impact curve.

**Results:**

Five independent predictive variables for HCC onset and two general characteristics of patients (age and gender) were incorporated into the score model. ROC and DCA curves showed that the score model had better predictive performance in discrimination and clinical net benefit compared with single variable or other score systems, with the area under the curve (AUC) of 0.890 (95% CI 0.856–0.925) in Changzhou cohort and 0.799 (95% CI 0.751–0.849) in Wuxi cohort. Meanwhile, stratification analysis indicated that the score model had good predictive values for patients with early tumor stage (AJCC stage I) or small tumors (< 2 cm). Moreover, the score of HCC patient began to increase at 30 months before clinical diagnosis and reach a peak at 6 months.

**Conclusion:**

Based on this model, we could optimize the current risk stratification at an early stage and consider further intensive surveillance programs for high-risk patients. It could also help clinicians to evaluate the progression and predict the prognosis of HCC patients.

**Supplementary Information:**

The online version contains supplementary material available at 10.1186/s12876-022-02489-2.

## Introduction

Hepatocellular carcinoma (HCC), one of the most fatal malignant tumors, is the third leading cause of cancer-related death in the world (8.2%) [1]. According to the *Global Cancer Statistics 2018* [2], the incidence and mortality rate of HCC in China after age standardization were 17.7/100,000 and 16.4/100,000, respectively. More than 70% of the patients are initially diagnosed at intermediate-to-advanced stages, which may be attributed to the insidious onset of HCC. Although medical technology has been improved greatly in recent years, the prognosis of patients with HCC remains unfavorable [3], the 5-years survival rate is only 11.7–14.1% [4]. In contrast, the 5-years survival rates of patients with early-stage who underwent the curative surgical therapies were approximate 69.0–86.2% [5]. As a result, developing a scientific screening method for early identification and timely treatment is critical for improving the prognosis of HCC patients.

Although the routine clinical screening methods for early HCC diagnosis historically involved abdominal ultrasound (US) examination and serum alpha-fetoprotein (AFP) detection [6]. However, the accuracy of the results obtained from the US is significantly influenced by the skills of the image observer, the conditions of instrumentation, and the characteristics of the patient (e.g., obesity, liver texture), with only 39–65% sensitivity for small liver tumors (less than 2 cm) [7]. AFP, a marker commonly used in HCC diagnosis, is positively correlated with the tumor size, but it remains negative in approximately 15–30% of HCC patients [8]. In addition, the AFP level was elevated significantly under certain pathological conditions, such as chronic liver disease, germ cell tumors, and gastric cancer [9, 10]. Recently, it has been reported that AFP-L3 and protein induced by Vitamin K absence or Antagonist-II (PIVKA-II) have high specificity in the diagnosis of HCC (92.9% and 89%, respectively), but the sensitivity of these individual serum markers for early HCC diagnosis is suboptimal [11, 12]. Furthermore, combined indicators such as neutrophil–lymphocyte ratio (NLR) [13], gamma-glutamyl transpeptidase to platelet ratio (GPR) [14], and gamma-glutamyl transpeptidase to lymphocyte ratio (GLR) [15] can be used to predict the prognosis and onset of HCC. Some studies have developed score models based on different variables such as gender, age, AFP levels, and pathological data, which have improved the accuracy of early HCC diagnosis. Nevertheless, some variables incorporated in the model were difficult to collect, which limits the usefulness of these models. Therefore, the models that could accurately predict HCC and conveniently guide clinicians for early HCC diagnosis were urgently needed. In this study, we retrospectively analyzed the levels of pre-diagnostic serum markers in patients with HCC to construct a non-invasive predictive model which could accurately predict the HCC onset and might facilitate early clinical detection and prognostic assessment.

## Material and methods

### Study subjects

We retrospectively reviewed the clinical data of 157 HCC patients hospitalized in Third People’s Hospital of Changzhou between June 2019 and December 2020, of whom 65 HCC patients had complete prognostic data. The inclusion criteria for our study were as follows: (1) Clinical diagnostic criteria for all HCC were based on the China guidelines (2017 Edition) [16] for *Diagnostic and Treatment of Primary Liver Cancer*; (2) The clinical data, including general characteristics, the surveillance time, and the levels of serum markers, were collected from all patients; (3) Patients with HCC had no other types of malignant tumors. Meanwhile, 734 patients suffering from chronic hepatitis or liver cirrhosis were enrolled as control. Additionally, clinical data of 305 patients (182 HCC patients and 123 liver cirrhosis patients) hospitalized in the Fifth People's Hospital of Wuxi during the same period were collected as external validation data, of whom 99 HCC patients had complete prognostic data.

### Serum variables analysis

The variables used to conduct the study are presented: (1) General clinical characteristics of all patients were collected, including age, gender, and medical history; (2) All the serum variables or combined indicators that could predict the onset of HCC were carefully screened in the PubMed database with the search term ‘serum predict HCC’. Then, 25 variables that could predict the early onset or prognosis of HCC were retrieved, including AFP, AFP-L3, PIVKA-II, Alanine transaminase (ALT), Aspartate transaminase (AST) levels, Albumin (ALB), Total protein (TP), Total bilirubin (TB), Apolipoprotein1, Antithrombin III, Fibrinogen, Neutrophil to lymphocyte ratio (NLR), Gamma-glutamyl transpeptidase (GGT) to platelet ratio (GPR), GGT to lymphocyte ratio (GLR), platelet to lymphocyte ratio (PLR), and bilirubin to albumin ratio (TB/ALB); (3) Predictive models for HCC constructed by other researchers, including GALAD model [17], ALBI score [18], and Fib-4 index [19]; (4) Follow-up data of patients were obtained either through the outpatient clinic or via telephone inquiry every 3 months.

### Statistical analysis

IBM SPSS software (version 22) was employed for statistical analysis. Continuous measurement data with normal distribution were expressed as mean ± standard deviation (${\overline{\text{x}}}$ ± SD) and a comparison of the two groups was done using a t-test. On the other hand, we presented the continuous measurement data with non-normal distribution as median (interquartile range) [*M* (*P*_25_, *P*_75_)] and non-parametric U-test (Mann–Whitney U test) was employed to compare the two groups of parameters. Categorical variables were described as percentages and the differences were compared using chi-square test. R (version 3.6.1) software was used for PSM analysis, ROC curve analysis, Logistic regression, and Decision curve analysis. Comparisons between the ROC curves were done using the method of Delong et al. [20]. Moreover, all tests were two-sided, and two-tailed test with *p* value < 0.05 was considered a significant difference.

## Results

### Baseline characteristics of patients

Totally 891 patients who met the entry criteria were enrolled in current study, including 157 patients with HCC (HCC group), and 734 patients with chronic hepatitis or liver cirrhosis (control group). Baseline analysis demonstrated that there were significant differences in patients’ general characteristics (age and gender) between the two groups (Table 1). Patients in the HCC group were older than those in the control group (59.66 ± 10.71 versus 54.23 ± 13.66, *p* < 0.001) and the male gender occupied a higher ratio (83.4% versus 62.5%, *p* < 0.001). To reduce the influence of these potential confounding factors, we performed PSM analysis with a 1:1 ratio and caliper of 0.05 using nearest neighbor method. After PSM, the final sample size in this study was 154 patients in each group, and most of the HCC patients were early stage (AJCC TNM Stage I–IV, 48.7%, 19.48%, 17.53%, 14.29%, respectively). The general characteristics (age, gender, and surveillance time) of the two groups were significantly balanced (*p* > 0.01), suggesting the PSM analysis improved the comparability of the two groups. However, statistical analysis of the matched data revealed that there were still differences in some serum variates between the two groups, such as AFP, AFP-L3, PIVKAII, etc. (*p* < 0.01).

**Table 1:** Baseline characteristics of patients in Changzhou cohort

Characteristics	Before PSM		*p* value	After PSM		*p* value
	HCC group (n = 157)	Control group (n = 734)		HCC group (n = 154)	Control group (n = 154)	
Age, ${\overline{\text{x}}}$ ± SD, (year)	59.66 ± 10.71	54.23 ± 13.66	< 0.001	59.33 ± 10.55	58.74 ± 12.35	0.652
Gender, male, (n, %)	131 (83.4)	459 (62.5)	< 0.001	128 (83.1)	131 (85.1)	0.755
Surveillance time, (day)	119.56 ± 162.93	131.44 ± 230.57	0.540	121.40 ± 163.97	123.54 ± 232.79	0.926
HBsAg, positive, (n, %)	126(80.8)	367 (50)	< 0.001	127 (82.5)	80 (51.9)	< 0.001
HBeAg, positive, (n, %)	7(4.5)	131 (17.9)	< 0.001	7 (4.5)	30 (19.5)	< 0.001
*Liver function*						
ALT, U/L	31.4 (20.65, 51)	84 (29, 284)	0.000	31.25 (20.65, 51)	57.9 (26, 246)	0.000
AST, U/L	36 (22.5, 61.5)	63 (31, 168)	0.000	35.5 (22, 61.5)	54 (29, 149)	< 0.001
Albumin, g/L	40.6 (35.8, 44)	39.25 (34.2, 43.3)	0.024	40.8 (35.85, 44)	38.8 (34.2, 42.4)	0.008
ALP, U/L	110 (81, 154.5)	109 (82, 153)	0.857	108.5 (80.5, 153.5)	113 (82, 152)	0.764
GGT, U/L	66 (33.6, 156.7)	99.9 (45.4, 207)	< 0.001	65.35 (32, 154.85)	115 (46.5, 206)	< 0.001
Total protein, g/L	68.8 (64.4, 72.55)	68.6 (63.5, 73.3)	0.799	68.9 (64.65, 72.6)	68.1 (62.1, 72.8)	0.311
Total bilirubin, umol/L	17.1 (12.75, 25.5)	18.95 (13.2, 33.3)	0.049	17.4 (12.85, 25.7)	18.2 (13.7, 38.2)	0.092
Prothrombin time, s	13.9 (13.2, 14.7)	13.9 (12.9, 15.3)	0.555	13.9 (13.2, 14.7)	13.9 (13.1, 15.5)	0.897
*Blood routine*						
WBC, 10^9^/L	4.94 (3.89, 6.15)	4.71 (3.71, 6.25)	0.644	4.85 (3.83, 6.14)	4.87 (3.81, 6.38)	0.954
Platelet, 10^9^/L	140 (91.5, 195)	149 (91, 205)	0.020	136.5 (90.5, 191)	135 (90, 199)	0.307
Neutrophil, 10^9^/L	3.03 (2.31, 3.93)	2.7 (1.94, 3.7)	0.024	3.01 (2.30, 3.84)	2.84 (2.05, 3.84)	0.367
Lymphocyte, 10^9^/L	1.22 (0.84, 1.70)	1.4 (1.01, 1.85)	0.000	1.24 (0.84, 1.72)	1.32 (0.94, 1.77)	0.055
PDW, %	13.9 (12.5, 15.6)	14.1 (12.2, 16.7)	0.161	13.9 (12.5, 15.6)	14.6 (12.3, 17.3)	0.07
MPV, %	11.4 (10.5, 12)	11.5 (10.6, 12.4)	0.114	11.45 (10.5, 12)	11.5 (10.6, 12.5)	0.035
*Tumor markers*						
AFP, ng/ml	21 (3.05, 547.55)	3.5 (1.9, 12.5)	0.000	20.85 (3.05, 605.75)	4.3 (2.1, 17.4)	0.000
AFP-L3, %	10.9 (0.5, 47.3)	0.5 (0.5, 6.0)	0.000	10.85 (0.5, 45.55)	0.5 (0.5, 6.8)	0.000
PIVKAII, mAU/ml	118 (23.5, 3888)	15 (11, 21)	0.000	125 (23.5, 3888)	16 (12, 21)	0.000
Tumor AJCC stages* (n, %)						
Stage I	77 (49.04)	NA		75 (48.7)	NA	
Stage II	31 (19.75)	NA		30 (19.48)	NA	
Stage III	27 (17.20)	NA		27 (17.53)	NA	
Stage IV	22 (14.01)	NA		22 (14.29)	NA	

*NA* Not applicable, *AJCC Stages The eighth edition American Joint Committee on Cancer (AJCC) TNM staging system, *ALP* Alkaline phosphatase, *GGT* Gamma-glutamyl transferase, *PDW* Platelet volume distribution width, *MPV* Mean platelet volume

### Construction of a predictive model for early onset of HCC based on the screened serum markers

ROC curve analysis was applied to evaluate the predictive efficiency of the serum markers, and univariate logistic regression analysis was used to screen risk factors for predicting HCC. Using the criteria of AUC > 0.55 and *p* < 0.25, respectively. 15 variates that could predict the onset of HCC were identified, including AFP, AFP-L3, AG, ALB, ALT, AST, GGT, TBIL, LY, PDW, MPV, GPR, TB/ALB, the status of HBsAg and HBeAg (Table 2; Additional file 1: Fig. S1). Multivariate logistic regression analysis revealed that AFP- L3, ALB, ALT, the status of HBsAg, and the status of HBeAg were independent risk factors for the onset of HCC. Finally, a score model for predicting the onset of HCC was constructed based on these independent risk factors and two general characteristics (age and gender) of patients: Score model = 0.009755*AFPL3 − 0.141957*Gender − 0.017824*Age (years) + 0.036840*ALB (g/L) − 0.004326*ALT (U/L) + 0.642490*(status of HBsAg) − 0.832696*(status of HBeAg), (Male equal to 1 and Female equal to 0; the status of HBsAg-positive and HBeAg-positive both equal to 1 and the status of HBsAg-negative and HBeAg-negative both equal to 0).

**Table 2:** ROC curve analysis and univariate logistic regression analysis of predictors for HCC onset

Variables	ROC curves				Logistic regression		
	AUC	Cutoff	Sensitivity	Specificity	OR	95% CI	*p* value
Gender	0.510	Female	0.169	0.851	0.88	0.58–1.35	0.565
Age	0.509	58.50	0.545	0.519	1.01	0.99–1.02	0.469
ALT	0.683	95.35	0.929	0.422	0.99	0.99–1.00	0.001
AST	0.647	51.50	0.714	0.552	1.00	0.99–1.00	0.005
GGT	0.609	72.35	0.578	0.656	1.00	1.00–1.00	0.006
Albumin	0.588	43.55	0.331	0.831	1.03	1.00–1.06	0.045
Total bilirubin	0.555	27.50	0.786	0.377	1.00	0.99–1.00	0.123
Alb/Glo	0.585	1.25	0.734	0.403	1.44	0.97–2.14	0.072
Lymphocyte	0.563	1.005	0.403	0.740	0.78	0.60–1.00	0.048
PDW	0.571	16.15	0.819	0.364	0.93	0.88–0.99	0.021
MPV	0.561	11.85	0.696	0.455	0.87	0.76–1.00	0.054
AFP	0.659	29.70	0.474	0.851	1.00	1.00–1.00	0.058
AFP-L3	0.687	13.50	0.461	0.955	1.02	1.01–1.02	0.000
TB/ALB	0.578	0.754	0.786	0.370	0.83	0.68–1.01	0.069
GPR	0.588	0.450	0.461	0.727	0.92	0.84–1.02	0.122
HBsAg	0.649	Positive	0.818	0.481	3.00	1.99–4.53	0.000
HBeAg	0.575	Negative	0.955	0.195	0.32	0.15–0.68	0.003

*GGT* Gamma-glutamyl transferase, *Alb/Glo* The ratio of albumin to globulin, *PDW* Platelet volume distribution width, *MPV* Mean platelet volume, *TB/ALB* The ratio of total bilirubin to albumin, *GPR* Gamma-glutamyl transpeptidase to platelet ratio

### Evaluation of the risk score model in Changzhou cohort

**Fig. 1:**
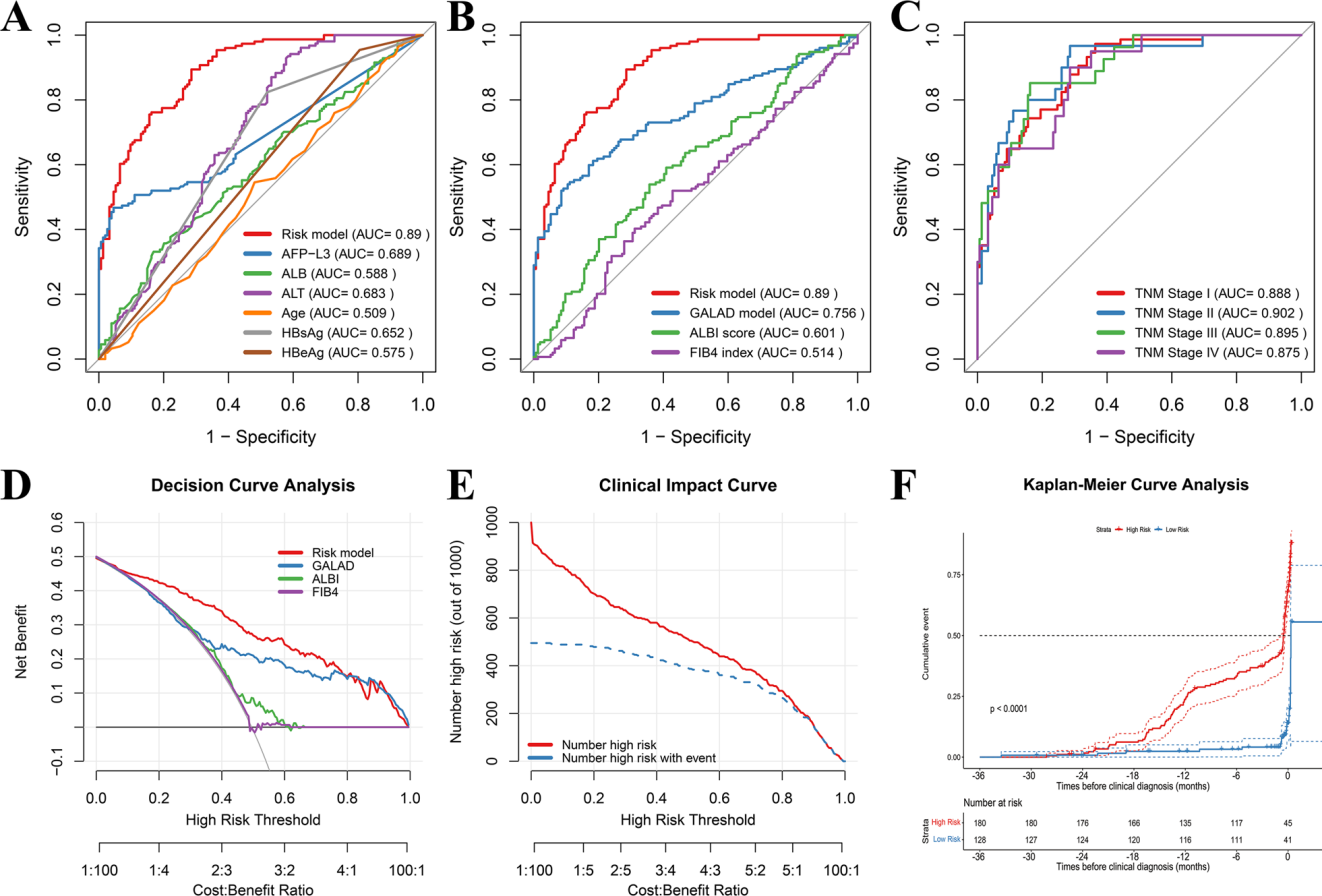
Performance of non-invasive predictive model for predicting the onset of HCC in Changzhou cohort. **A** ROC curve analysis of the risk model and single variable for predicting the onset of HCC. **B** ROC curve analysis of the risk model and other score systems for predicting the onset of HCC. **C** ROC curve analysis of the risk model for predicting the onset of HCC in patients with different AJCC TNM stages. **D** Decision curve analysis demonstrates the clinical net benefit of the risk score model and other score systems for the onset of HCC in Changzhou cohort. **E** Clinical impact curve of the risk model. **F** Cumulative event between high risk and low risk groups at indicated time before clinical diagnosis

According to the score model, the score for each patient in Changzhou cohort was calculated. ROC curve of the risk score model showed that the AUC value for predicting the onset of HCC was 0.890, which was significantly higher than that of individual characteristics (0.509–0.689, *p* < 0.05; Fig. 1A; Additional file 2: Table S1), ALBI score, Fib-4 index, and GALAD model (0.514–0.756, *p* < 0.05; Fig. 1B; Additional file 2: Table S1). Interestingly, the risk score model also had similar high predictive values in patients with different TNM stages (AUC: 0.875–0.902, Fig. 1C), but no remarkable differences among them (*p* > 0.05, Additional file 2: Table S2). Meanwhile, decision curve analysis revealed that this score model had a larger clinical net benefit compared with the GALAD model, ALBI score, and Fib-4 index (Fig. 1D). Moreover, clinical impact curves also showed that this risk model had good prediction power for the onset of HCC (Fig. 1E). Furthermore, the Kaplan–Meier curve was performed. For Changzhou cohort, the cumulative incidence of HCC was significantly higher in patients with high-risk score than in those with low-risk score (*p* < 0.05, Fig. 1F). In addition, the score predictive model showed promising levels of sensitivity (89.4% versus 33.1–82.4%) and NPV (87.3% versus 55.4–73.3%), respectively (Additional file 2: Table S1), even in patients with different tumor stages (sensitivity: 85.2–97.3%; NPV: 97.0–99.1%) (Additional file 2: Table S2). In conclusion, this risk score model based on the pre-diagnostic level of serum markers was a reliable and superior model for predicting the onset of HCC.

### Evaluation of the model score in HCC progression

**Fig. 2:**
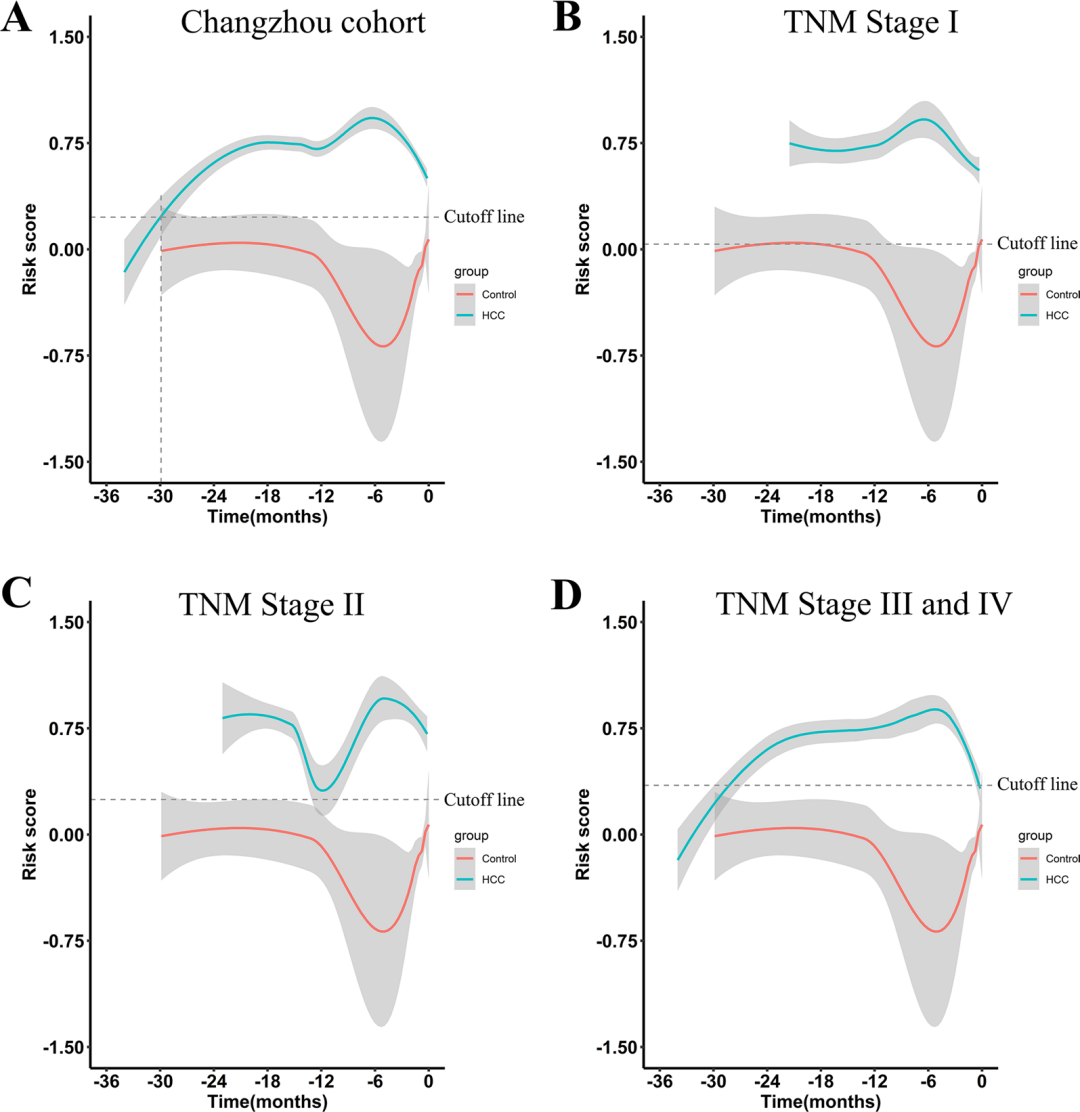
The longitudinal changes of the risk score for disease progression. **A**–**D** The smooth curve of risk score in selected datasets (**A** Changzhou cohort. **B** Patients with AJCC TNM stage I. **C** Patients with AJCC TNM stage II. **D** Patients with AJCC stage III and IV)

To investigate the potential clinical value of this model in the progression of HCC, we calculated the risk score of patients at each clinical surveillance time and visualized the longitudinal changes of risk score for disease progression. The smooth curve showed that the risk score of the HCC group before clinical diagnosis time was greatly elevated than that of the control group. Interestingly, the risk score of the HCC group increased at 30 months before clinical diagnosis and reached a peak at 6 months, suggesting that the score model may be a useful tool for early predicting the risk of HCC (Fig. 2A). Furthermore, similar trends were also observed in patients with different tumor stages (Fig. 2B–D). In addition, according to the best cutoff value for predicting the onset of HCC (0.227), patients were divided into two subgroups (high-risk and low-risk subgroups). The Kaplan–Meier curve analysis revealed that patients in the high-risk subgroup had a lower overall survival (*p* < 0.05, Additional file 1: Fig. S2A). These results strongly indicated that the risk score model could predict the onset of HCC at early time points and might facilitate the prognostic evaluation of HCC patients.

### Validation of the risk model in Wuxi cohort

**Fig. 3:**
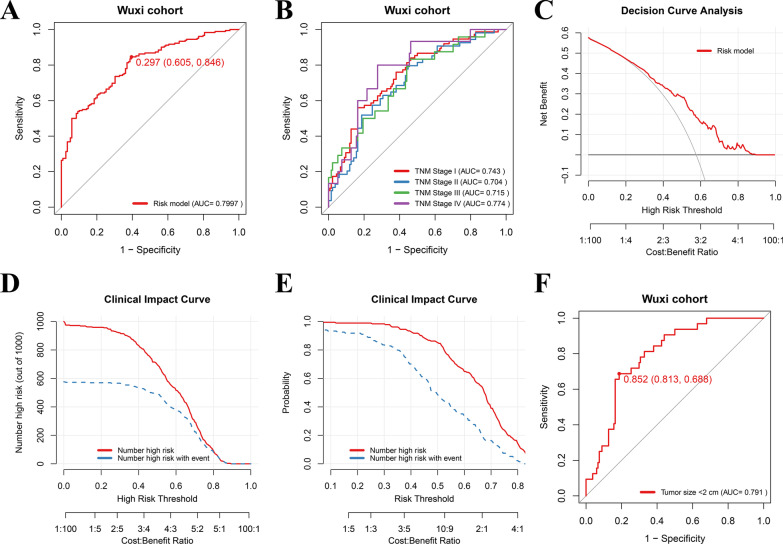
Validation of the risk model in Wuxi cohort. **A** Performance of the risk model for predicting the onset of HCC. **B** Performance of the risk model for predicting the onset of HCC in patients with different AJCC TNM stages. **C** Decision curve analysis demonstrates the clinical net benefit of the risk score model for the onset of HCC. **D**–**E** Clinical impact curve of the risk model. **F** Predictive value of the risk model for the onset of HCC in patients with small tumor (< 2 cm)

Since the risk score model based on the non-invasive serum markers showed reliable and superior predictive ability for HCC development in Changzhou cohort, we further validated the predictive ability of the risk score model in an independent cohort from Wuxi (Additional file 2: Table S3), and the score for every patient was calculated using the same formula. ROC curve analysis was performed for predicting the development of HCC. The AUC value was 0.799 with a sensitivity of 84.6% and specificity of 60.5%, which demonstrated that this score model still had a superior predictive ability (Fig. 3A). Furthermore, the risk model also demonstrated strong predictive power for patients with different tumor stages based on AJCC criteria (AUC, 0.704–0.774, Fig. 3B). Decision curve analysis and clinical impact curve revealed that the risk score model had a good clinical utility for predicting the onset of HCC in Wuxi cohort (Fig. 3C–E). In addition, the score model also had a high predictive value for small tumor (diameter size < 2 cm, AUC, 0.791, Fig. 3F). However, survival analysis showed no difference between two subgroups (*p* > 0.05, Additional file 1: Fig. S2B). In summary, the risk score model constructed using non-invasive serum markers was a reliable and effective tool for predicting the development of HCC.

## Discussion

The prognosis of the HCC patient remains dismal due to the insidious onset [21]. Effective early screening strategies and curative treatments could significantly improve the prognosis of HCC patients [22]. However, Current approaches and biomarkers have some limitations in the early HCC diagnosis [23], such as poor compliance, low sensitivity, and time consumption. Surveillance of early-stage patients is inadequate. On the other hand, Early and accurate HCC detection with non-invasive biomarkers remains an unmet clinical need. In this study, we screened the pre-diagnostic serum biomarkers related to the development of HCC and constructed a non-invasive risk score model for early detection of HCC. This score model showed satisfactory discriminant function (AUC: 0.890, with 89.4% sensitivity and 71.4% specificity, respectively). Similar predictive value of the model for HCC onset was observed in an independent cohort (AUC: 0.799). Moreover, the predictive efficacy of the risk model showed powerful and satisfactory clinical performance, which was evaluated by the Kaplan–Meier curve, decision curve analysis, and clinical impact curve analysis. Notably, the risk score model still showed satisfactory predictive power in patients with early stage (AJCC Stage I) or those with small tumors (< 2 cm). Based on this model, we could optimize the early risk stratification of patients and evaluate the prognosis of HCC patients.

This model consists of five pre-diagnostic serum biomarkers, and these markers could be grouped into three categories: 1) host factors (age, gender, and ALB); 2) viral activity-related factors (ALT, HBsAg status, and HBeAg status); 3) malignant hepatocytes growth-related factors (AFP-L3). Compared to the GALAD model, the indicators of viral activity-related factors were included. Hepatitis B virus (HBV) infection is a key risk factor for the etiology of HCC, especially in eastern Asia and sub-Saharan Africa [24], nearly 90% of HCC patients in China had a history of HBV infection [1]. Several mechanisms by which Hepatitis B virus progression to HCC were proposed [25]. Nevertheless, HBeAg positivity indicated active replication of HBV virus [26]. and several case–control studies indicated that HBeAg seemed to be a better predictive marker for HBV-related HCC [27]. AFP and AFP-L3 are the most widely used serum markers in HCC diagnosis. Studies have demonstrated that serum AFP levels in HCC patients were elevated with disease progression and correlated with tumor size [28], the sensitivity and specificity of AFP for early detection of HCC were 45.3–62% and 87–93%, respectively [29]. AFP isoform L3 (AFP-L3), a glycoprotein of primary origin in hepatocellular cancer cells, was a marker independent of AFP. The levels of AFP-L3 were significantly associated with the development of HCC, with a sensitivity of 45.9–50.7% and a specificity of 92.9%. Nearly 34.3% of HCC patients with normal AFP levels had abnormal AFP-L3 expression before HCC diagnosis [11]. Recent studies have reported that the serum AFP-L3 could be detected in about 35% of patients with small HCC [30]. In addition, serum level of ALB, an essential indicator of nutrition, reflects the capability to regulate the immune and antioxidant reaction against carcinogenesis [31, 32]. Several studies have reported that the abnormal levels of ALB could independently predict a worse prognosis in patients with HCC [33]. Taken together, these markers, included in our score model, are related to the development and prognosis of the tumor. Therefore, this scoring model we constructed could be applied to numerous objectives for HCC, such as stratification of risk, follow-up care after treatment, and prediction of prognosis.

There are still certain limitations. First, the total number of HCC patients in our study is inadequate. Second, the follow-up data for all patients obtained from hospital records were partially missing, especially for patients with early-stage HCC (AJCC stage I and II). Finally, the genotypes of virus were not incorporated into the score model. Therefore, we will enlarge the patient cohort, long-term follow-up, and multi-center studies in the future to further validate the clinical values of the risk model.

## Conclusion

In summary, the score model established in this study had the characteristics of high accuracy, reliable, and easy to use. Patients with high-risk scores should consider more intensive surveillance programs. Prospectively, clinicians could use this risk score model as early as possible to screen high-risk patients, evaluate the progression and predict the prognosis of HCC patients.

## Supplementary Information

**Additional file 1. Figure S1.** The Venn diagram indicated intersection of the serum markers associated with HCC onset (Green, serum markers identified by ROC analysis with AUC > 0.55; blue, serum markers screened by univariate logistic regression analysis with *p* < 0.25). **Figure S2.** Kaplan–Meier analysis for overall survival between high risk group and low risk group. **A** In Changzhou cohort. **B** In Wuxi cohort.

**Additional file 2. Table S1.** Predictive accuracy comparison of the variables for the onset of HCC in Changzhou cohort. **Table S2.** Predictive accuracy comparison of score model for the onset of HCC in patients with different AJCC TNM stages. **Table S3.** Baseline characteristics of patients in Wuxi cohort.

## Data Availability

All data generated and/or analyzed during this study are available from the corresponding author on reasonable request.

## Abbreviations

HCC: Hepatocellular carcinoma
ROC: Receiver operating characteristic
K–M curve: Kaplan–Meier curve
DCA: Decision curve analysis
AUC: Area under the curve
95%CI: 95% Confidence interval
AFP: Alpha-fetoprotein
AFP-L3: Lens culinaris agglutinin-reactive fraction of AFP
PIVKA-II: Protein induced by Vitamin K absence or Antagonist-II
ALB: Albumin
ALP: Alkaline phosphatase
GGT: Gamma-glutamyl transpeptidase
TP: Total protein
WBC: White blood cell
NEUT: Neutrophil count
NLR: Neutrophil–lymphocyte ratio
GPR: GGT to platelet ratio
GLR: GGT to lymphocyte ratio
