# Supplementary material for: Construction and validation of a predictive model for hepatocellular carcinoma based on serum markers
Source: BMC Gastroenterol. 2022 Sep 13;22:418. doi: 10.1186/s12876-022-02489-2 (PMC9472335; doi:10.1186/s12876-022-02489-2)
Supplement: Supplementary file 1 — Additional file 1. Figure S1. The Venn diagram indicated intersection of the serum markers associated with HCC onset (Green, serum markers identified by ROC analysis with AUC > 0.55; blue, serum markers screened by univariate logistic regression analysis with p < 0.25). Figure S2. Kaplan–Meier analysis for overall survival between high risk group and low risk group. A In Changzhou cohort. B In Wuxi cohort. [file 12876_2022_2489_MOESM1_ESM.docx]

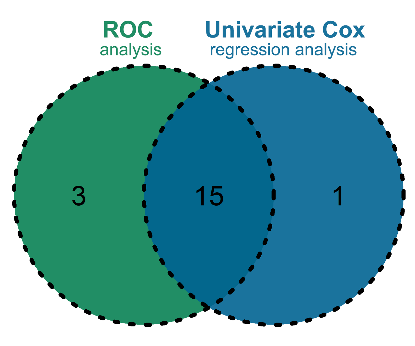


**Supplementary Figure 1.** The Venn diagram indicated intersection of the serum markers associated with HCC onset (Green, serum markers identified by ROC analysis with AUC>0.55; blue, serum markers screened by univariate logistic regression analysis with p<0.25).


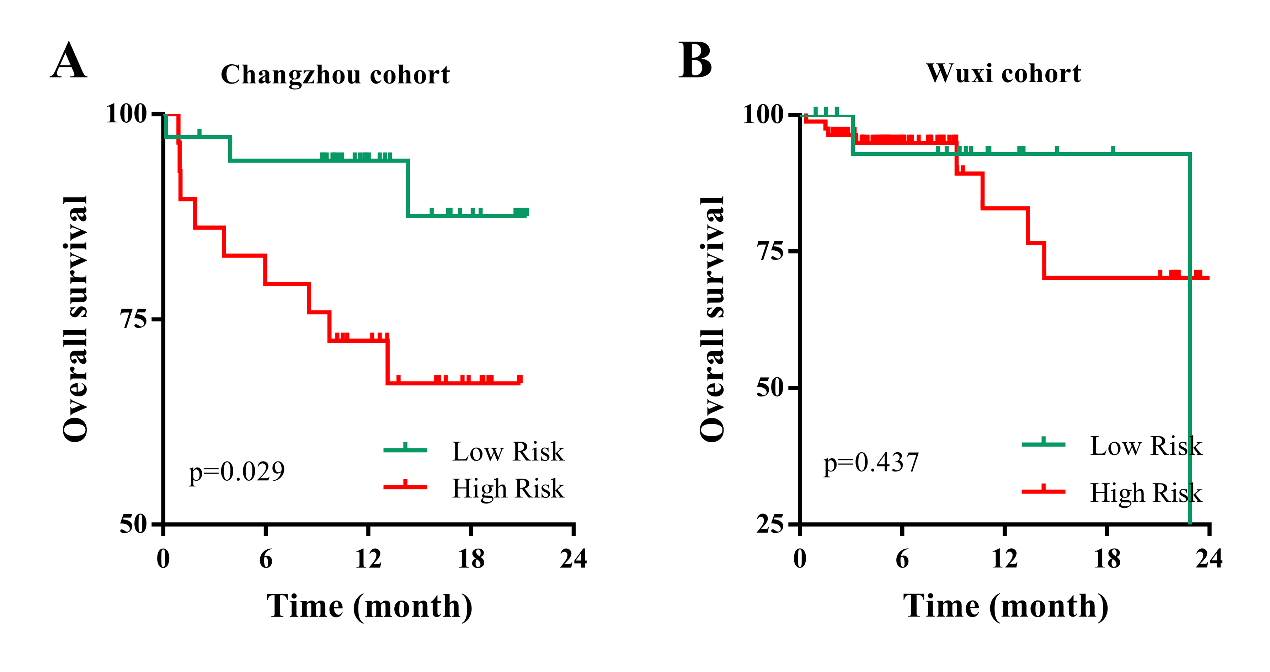


**Supplementary Figure 2.** Kaplan-Meier analysis for overall survival between high risk group and low risk group. A. In Changzhou cohort. B. In Wuxi cohort.
