# Supplementary material for: Construction and validation of a predictive model for hepatocellular carcinoma based on serum markers
Source: BMC Gastroenterol. 2022 Sep 13;22:418. doi: 10.1186/s12876-022-02489-2 (PMC9472335; doi:10.1186/s12876-022-02489-2)
Supplement: Supplementary file 2 — Additional file 2. Table S1. Predictive accuracy comparison of the variables for the onset of HCC in Changzhou cohort. Table S2. Predictive accuracy comparison of score model for the onset of HCC in patients with different AJCC TNM stages. Table S3. Baseline characteristics of patients in Wuxi cohort. [file 12876_2022_2489_MOESM2_ESM.docx]

**Supplementary Table 1.** Predictive accuracy comparison of the variables for the onset of HCC in Changzhou cohort.

| Variables | Cutoff value | AUC | 95% CI | p value* | Sensitivity (%) | Specificity (%) | PPV (%) | NPV (%) |
| --- | --- | --- | --- | --- | --- | --- | --- | --- |
| AFP-L3, % | 13.5 | 0.689 | 0.632-0.746 | 1.095e-09 | 46.7 | 95.5 | 91 | 64.5 |
| ALB, g/l | 43.55 | 0.588 | 0.524-0.651 | 3.829e-21 | 33.1 | 83.1 | 66.2 | 55.4 |
| HBsAg | positive | 0.652 | 0.602-0.702 | 4.684e-22 | 82.4 | 48.1 | 61.2 | 73.3 |
| GALAD | 2.905 | 0.756 | 0.701-0.811 | 2.963e-05 | 54.6 | 88.9 | 83 | 66.3 |
| Risk model | 0.227 | 0.890 | 0.856-0.925 | - | 89.4 | 71.4 | 75.4 | 87.3 |

AUC, area under curve; CI, confidence interval; PPV, positive predictive value; NPV, negative predictive value; *, compared to the risk model.

**Supplementary Table 2.** Predictive accuracy comparison of score model for the onset of HCC in patients with different AJCC TNM stages.

| TNM Stage | Cutoff value | AUC | 95% CI | p value* | Sensitivity (%) | Specificity (%) | PPV (%) | NPV (%) |
| --- | --- | --- | --- | --- | --- | --- | --- | --- |
| Stage I | 0.037 | 0.888 | 0.846-0.930 | - | 97.3 | 63.6 | 56.2 | 98.0 |
| Stage II | 0.247 | 0.902 | 0.844-0.960 | 0.621 | 96.7 | 71.4 | 39.7 | 99.1 |
| Stage III | 0.508 | 0.895 | 0.836-0.955 | 0.766 | 85.2 | 83.8 | 47.9 | 97.0 |
| Stage IV | 0.251 | 0.875 | 0.803-0.947 | 0.589 | 90.0 | 71.4 | 29.0 | 98.2 |

AUC, area under curve; CI, confidence interval; PPV, positive predictive value; NPV, negative predictive value; *, compared to the patients with tumor TNM stage I.

**Supplementary Table 3.** Baseline characteristics of patients in Wuxi cohort.

| Characteristics | HCC group (n=182) | Control (n=123) | p value |
| --- | --- | --- | --- |
| Age,‾x±SD, (year) | 61±11.03 | 57±12.59 | 0.069 |
| Gender, male, (n, %) | 134 (73.63) | 68 (55.28) | 0.001 |
| ALB, g/L | 39.3 (35.1, 43.7) | 37.9 (33.1, 43.6) | 0.151 |
| ALP, U/L | 106.6 (81, 154) | 109 (76, 147) | 0.385 |
| AFP-L3, % | 15 (0.5, 58.4) | 0.5 (0.5, 6.2) | 0.000 |
| AJCC TNM stage^*^ (n, %) |  |  |  |
| Stage I | 75 (41.21) | NA |  |
| Stage II | 54 (29.67) | NA |  |
| Stage III | 24 (13.19) | NA |  |
| Stage IV | 15 (8.24) | NA |  |
| Missing | 14 (7.69) | NA |  |

NA: Not applicable; ^*^AJCC Stages: The eighth edition American Joint Committee on Cancer (AJCC) TNM staging system.
